# Research on adolescents' digital sports participation behavior based on the UTAUT2 model: the mediating role of virtual-real integration

**DOI:** 10.3389/fpsyg.2026.1742537

**Published:** 2026-03-03

**Authors:** Jiying Wu, Bowen Zhang

**Affiliations:** 1 School of Management and Journalism, Shenyang Sport University, Shenyang, China; 2 Department of Physical Education, Northeastern University, Shenyang, Liaoning, China

**Keywords:** adolescents, digital sports participation, structural equation modeling, UTAUT2, virtual-real integration

## Abstract

**Introduction:**

Drawing on the Unified Theory of Acceptance and Use of Technology 2 (UTAUT2) and technological affordances theory, this study constructs a theoretical model to investigate key factors influencing adolescents' digital sports participation and its underlying mechanisms. With virtual-real integration as the core mediating variable, we examine how five antecedents—performance expectancy, effort expectancy, social influence, facilitating conditions, and hedonic motivation—affect active and deep sports participation through this mediator.

**Methods:**

Data were collected from 417 adolescent participants using validated scales. Structural equation modeling was employed to test the proposed relationships, including direct and mediating effects.

**Results:**

The results confirm that virtual-real integration serves as a critical behavioral mechanism linking technology affordances to digital sports participation. The UTAUT2 core variables demonstrate universal explanatory power, though their influence pathways diverge. Specifically, performance expectancy's effect on deep participation is fully mediated by virtual-real integration; facilitating conditions has no direct effect on either participation behavior, with its influence entirely mediated through virtual-real integration. The model explains 50.7%, 51.5%, and 43.1% of the variance in virtual-real integration, active participation, and deep participation, respectively, indicating strong explanatory power.

**Discussion:**

By introducing virtual-real integration as a behavioral construct, this study extends the theoretical boundaries of UTAUT2, providing explanations and empirical evidence for the transformation process from technology acceptance to deep sports participation among adolescents. The findings offer insights for designers, educators, and policymakers: prioritize features that foster virtual-real integration, leverage community influence to establish blended online-offline sports communities, and improve accessibility conditions to support integrated behaviors, thereby effectively promoting technology-integrated sports activities among adolescents.

## 1 Introduction

Amid the global wave of digitalization, adolescents, who are often described as “digital natives,” are engaging with digital technologies at an increasingly profound level. These technologies have permeated diverse fields such as education, entertainment, and social interaction, profoundly reshaping how young people learn and live. A particularly remarkable technology-driven transformation is occurring in the realm of sports participation. Digital sports technologies—exemplified by fitness apps (Angosto et al., 2023), motion-sensing games, smart wearable devices (Zhang et al., 2023; Wang et al., 2022), and virtual reality (VR) systems (Kim and Ko, 2019; Chen, 2024; Lee and Oh, 2022)—are significantly expanding the boundaries and forms of adolescent sports engagement by offering diverse virtual sports scenarios and real-time data feedback, providing new potential for addressing the global issue of insufficient physical activity among adolescents.

Against this backdrop, a thorough investigation into the mechanisms underlying adolescents' acceptance and use of such technologies has become essential for understanding and designing digital sports technologies that can effectively promote healthy behaviors among youth. The Technology Acceptance Model (TAM) has long been widely adopted as a foundational theoretical framework. Its core constructs—perceived usefulness (PU) and perceived ease of use (PEOU)—have been consistently validated as significant predictors of users' behavioral intentions toward sports technologies such as fitness trackers (Qian and Kim, 2025; Seong and Hong, 2022). (Seong and Hong 2022) applied the Technology Readiness and Acceptance Model (TRAM) to virtual sports such as screen golf, confirming that perceived usefulness is the most critical factor driving participation intention. (Lee and Oh 2022), building on TAM, further noted that besides perceived usefulness and ease of use, users' willingness to participate is also influenced by factors such as aesthetic experience, presence, and flow.

However, classical theories represented by TAM primarily focus on explaining the formation of “usage intention” and offer limited explanatory power for how such intention translates into specific—especially deep and sustained—participation behaviors. To more comprehensively capture the full process from “willingness to use” to “continuous and deep participation,” scholars have increasingly turned to more integrative theoretical frameworks. The Unified Theory of Acceptance and Use of Technology (UTAUT) and its consumer-oriented extension (UTAUT2) significantly improve the overall prediction of technology adoption intention and usage behavior by integrating multiple antecedents such as performance expectancy, effort expectancy, social influence, facilitating conditions, and hedonic motivation. Even so, the UTUAT2 model still exhibits a key theoretical gap: it does not sufficiently reveal the specific psychological or behavioral processes through which users virtual experiences and real world physical activities merge after technology acceptance and use, thereby fostering deep and sustained behavioral change. To address this gap and accurately depict the concrete process through which digital technology integrates into sports behavior, this study introduces “Virtual-Real Integration” as a core mediating construct. This construct delineates the critical pathway of transformation from technology use to deep behavioral internalization, with its core lying in how individuals proactively enhance and deepen their participation in real-world sports activities through the functionalities of digital technologies.

In summary, this study aims to develop an extended model that incorporates performance expectancy, effort expectancy, social influence, facilitating conditions, and hedonic motivation from UTAUT2 as antecedents, virtual-real integration as the key mediator, and active sports participation and deep sports participation as outcome variables. The research not only tests the applicability of the core UTAUT2 constructs in the context of adolescent digital sports but also focuses on revealing how these antecedents ultimately drive tangible and in-depth sports participation behaviors by stimulating and facilitating the core process of virtual-real integration. It is expected that this study will provide a more nuanced and explanatory theoretical framework and empirical evidence for understanding and designing digital sports technologies that can effectively promote healthy behaviors among adolescents.

## 2 Model construction and hypotheses

### 2.1 Theoretical foundations of the model

The Unified Theory of Acceptance and Use of Technology 2 (UTAUT2) is an expanded version proposed by (Venkatesh et al. 2012) based on the original Unified Theory of Acceptanceand Use of Technology (UTAUT) (Venkatesh et al., 2003), specifically targeting consumer technology acceptance behavior. This model includes seven core independent variables: performance expectancy, effort expectancy, social influence, facilitating conditions, hedonic motivation, price value, and habit. Existing empirical studies indicate that this model explains up to 70% of the variance in usage intention (Venkatesh et al., 2016). The UTAUT2 model is effective in explaining the willingness and behavior of different groups to adopt innovative technologies across various cultural environments and social contexts, and has become an important theoretical tool for predicting and analyzing the acceptance of information technology, virtual reality technology, etc., by individuals or organizations in learning contexts (Kosiba et al., 2022).

This study adopts UTAUT2 as its foundational framework. While this theory effectively predicts initial intention to adopt a technology, its explanatory mechanism for the internalization and transformation process—specifically, how intention translates into sustained and in-depth actual behavior—remains underdeveloped. To address this theoretical gap and align with the specific context of adolescents' digital sports participation, the model has been appropriately streamlined and enhanced by incorporating the theory of technological affordances as a core supplementary perspective, thereby strengthening its theoretical foundation.

First, the UTAUT2 model (Venkatesh et al., 2012) was contextualized and simplified for this study. We retained the five core antecedent variables—performance expectancy, effort expectancy, social influence, facilitating conditions, and hedonic motivation—which constitute the initial psychological drivers of technology adoption. Considering that adolescents primarily use free or low-cost applications and are still in the formative stage of behavioral patterns, the variables of “price value” and “habit” were excluded. This refinement allows the model to focus more closely on the key driving mechanisms during the early and middle stages of participation.

Second, and central to the theoretical innovation of this model, is the introduction of the key mediating variable: virtual–real integration. The conceptualization of this construct is grounded in the theory of technological affordances. Originating from Gibson's ecological psychology (Gibson, 1979), affordances refer to the possibilities for action that an object offers to an actor, with the realization of these possibilities closely tied to the actor's perceptual capabilities. The cognitive essence lies in understanding how users perceive and apply technological features—that is, any affordance must be perceived by the actor (Norman, 1988). In the fields of organization and information systems, technological affordances have been defined as the possibilities for action or change offered by information and communication technologies to individuals or organizations (Leonardi and Vaast, 2017), as well as the process of realizing these possibilities (Du et al., 2019). The theory emphasizes that the value of technology lies not in its inherent qualities, but in the dynamic relationship through which it is perceived and utilized by actors in specific contexts to create value. Based on this, virtual-real integration is operationally defined in this study as: the process through which adolescents actively perceive and utilize the possibilities for action provided by digital technologies during digital sports participation, systematically connecting and integrating them with real-world physical activities to create coherent behavioral value. It extends beyond the typical adoption or usage focus of UTAUT2, concretely representing the critical transformative pathway from technology acceptance to behavioral deepening. In other words, the antecedent variables not only directly influence behavior but also indirectly drive meaningful participation by fostering this deep integration.

Furthermore, to accurately capture different dimensions of behavioral outcomes, this study distinguishes between two outcome variables: active sports participation focuses on the external manifestation and quantitative engagement at the behavioral level, reflected in the proactiveness, frequency, planning, and self-management behaviors exhibited by individuals when using digital sports technologies. It captures the quantity and routinization of participation. Deep sports participation emphasizes the internal state and qualitative immersion at the experiential level. Its core characteristics include high emotional engagement, deep cognitive focus during physical activity, and the internalization of value and sense of accomplishment derived from sports, drawing theoretical grounding from flow theory. It reflects the quality and sense of meaning in the participation experience.

In summary, the theoretical model constructed in this study systematically examines how the UTAUT2 antecedent variables ultimately influence adolescents' sports participation—from behavioral expression to experiential depth—through the behavioral transformation mechanism of virtual–real integration. This work not only serves as an empirical test of UTAUT2 in the context of adolescent digital sports but also represents an important extension of its theoretical boundaries. It explicitly offers a testable mediating mechanism that explains how technology adoption leads to deep behavioral change, thereby directly addressing the framework's limitations in explaining behavioral transformation processes.

### 2.2 Research hypothesis

#### 2.2.1 Performance expectancy

Performance expectancy refers to the extent to which adolescents believe that using digital sports technology will help them enhance their sports performance and achieve exercise goals, such as losing weight, increasing endurance, or optimizing training results through technological assistance. As a key cognitive factor influencing user technology acceptance, performance expectancy has not only been widely validated to have a significant positive impact on continuance usage intention (Venkatesh et al., 2003; Yuan et al., 2015), but also, by strengthening adolescents recognition of the technology's value, further promotes their integration of offline physical activities with online digital functions. Specifically, when adolescents expect that digital technology can tangibly improve their exercise outcomes, they are more inclined to actively use these tools to record data from offline activities and adjust training plans based on online feedback, thereby deepening the level of virtual-real integration. Based on this, the following hypotheses are proposed:

H1a: Performance expectancy has a significant positive effect on adolescents active participation in sports.
H1b: Performance expectancy has a significant positive effect on adolescents deep participation in sports.
H1c: Performance expectancy has a significant positive effect on the degree of virtual-real integration.

#### 2.2.2 Effort expectancy

Effort expectancy refers to the degree of ease associated with adolescents use of digital sports technology. If adolescents find the relevant platforms or devices easy to operate and user-friendly, they are more likely to form positive expectations about the technology's utility, which in turn increases their willingness to actively engage and participate deeply in sports activities. Research indicates that effort expectancy notonly has a significant positive impact on users continuance usage intention (Venkatesh et al., 2003; Lee et al., 2017) but also, by lowering the barrier to technology use, encourages more frequent and in-depth connections between physical sports and digital tools. For example, they become more willing to use apps to record sports data, accept online guidance, and adjust offline training plans, thus reinforcing the virtual-real integration behavior pattern. Therefore, the following hypotheses are proposed:

H2a: Effort expectancy has a significant positive effect on adolescents active participation in sports.
H2b: Effort expectancy has a significant positive effect on adolescents deep participation in sports.
H2c: Effort expectancy has a significant positive effect on the degree of virtual-real integration.

#### 2.2.3 Social influence

Social influence refers to the extent to which adolescents perceive that significant others (e.g., peers, parents, or teachers) believe they should use digital sports technologies, and the resulting social pressure they experience during participation. According to the Theory of Reasoned Action, an individual's behavioral intention is significantly influenced by subjective norms. When adolescents social networks hold supportive or encouraging attitudes toward digital sports technology, it enhances their willingness to use technology to assist sports participation, thereby increasing the frequency and depth of involvement. In contrast, perceived negative evaluations or social pressure may inhibit their participation motivation. Existing research shows that social influence is a key variable predicting users continuance technology usage behavior. Furthermore, positive social identity and group modeling effects can prompt adolescents to more actively engage in virtual-real integrated sports participation. Accordingly, the following hypotheses are proposed:

H3a: Social influence has a significant positive effect on adolescents active sports participation.
H3b: Social influence has a significant positive effect on adolescents deep sports participation.
H3c: Social influence has a significant positive effect on the degree of virtual-real integration.

#### 2.2.4 Facilitating conditions

Facilitating conditions refer to adolescents perceptions of the resources and support available to facilitate their use of a digital sports technology system. This encompasses factors such as the accessibility of hardware devices, the stability of the network environment, the availability of operational guidance, and the compatibility of platforms with commonly used devices. According to technology acceptance research, when such support conditions are readily accessible, the technological barriers to participation are significantly reduced. This lowered barrier not only makes adolescents more inclined to participate actively and sustain engagement in sports practice but also helps promote the integration of online functions with offline sports activities. Studies show that facilitating conditions have a significant impact on users technology usage intention and actual behavior (Venkatesh et al., 2012; Du and Liang, 2024). Based on this, the following hypotheses are proposed:

H4a: Facilitating conditions have a significant positive effect on adolescents active sports participation.
H4b: Facilitating conditions have a significant positive effect on adolescents deep sports participation.
H4c: Facilitating conditions have a significant positive effect on the degree of virtual-real integration.

#### 2.2.5 Hedonic motivation

Hedonic motivation refers to the fun or pleasure derived from using a technology (Venkatesh et al., 2012). In the context of this study, it represents the enjoyment, pleasure, and sense of immersion experienced by adolescents when using digital sports technology. Such positive affective experiences from human-technology interaction are often distinctive and less attainable in traditional sports settings. When adolescents derive enjoyment from using virtual sports scenarios, gamified fitness applications, or smart wearable devices, their intrinsic motivation to participate in sports activities significantly increases, making them more inclined toward active and sustained deep engagement. This pleasurable experience not only directly enhances participation intention but also prompts adolescents to more actively establish connections between the online virtual environment and offline physical activities. For instance, they might persist with offline exercises to continue the fun derived from gamified sports, thereby strengthening virtual-real integration. Extant research has widely confirmed hedonic motivation as a key factor influencing users continuance usage behavior (Venkatesh et al., 2012; Tamilmani et al., 2019; Brown and Venkatesh, 2005). Based on this, the following hypotheses are proposed:

H5a: Hedonic motivation has a significant positive effect on adolescents active sports participation.
H5b: Hedonic motivation has a significant positive effect on adolescents deep sports participation.
H5c: Hedonic motivation has a significant positive effect on the degree of virtual-real integration.

#### 2.2.6 The mediating role of virtual-real integration

Based on the theory of technological affordances, this study proposes the mediating role of virtual–real integration in the adolescent sports participation model. The theory of affordances emphasizes that the value of technology lies in the possibilities for action it offers to users, as well as the process through which individuals perceive and realize these possibilities (Gibson, 1977; Norman, 1988). In the context of digital sports participation, adolescents actively perceive and utilize the various behavioral possibilities provided by digital technologies—such as data tracking, social connectivity, and gamified feedback—and systematically integrate virtual features with real-world physical activities, thereby forming consistent behavioral patterns.

Specifically, when adolescents use smart wearables to record exercise data, adjust training plans based on online feedback, or organize group activities through online platforms that translate into offline collaborative sports, the affordances offered by digital technology—such as recording affordance, feedback affordance, social affordance, and motivational affordance—are gradually perceived, utilized, and internalized. This process of virtual–real integration not only enhances the enjoyment of physical activities but also deepens adolescents' participation experiences through data-driven management and socially facilitated engagement. Based on the above mechanisms, the following hypotheses are proposed:

H6a: The degree of virtual-real integration has a significant positive effect on adolescents active sports participation.
H6b: The degree of virtual-real integration has a significant positive effect on adolescents deep sports participation.
H7–H11: The degree of virtual-real integration plays a mediating role in the relationships between Performance Expectancy (H7a/b), Effort Expectancy (H8a/b), Social Influence (H9a/b), Facilitating Conditions (H10a/b), and Hedonic Motivation (H11a/b) and the two types of sports participation (Active/Deep), respectively.

### 2.3 Theoretical model

**Figure 1:**
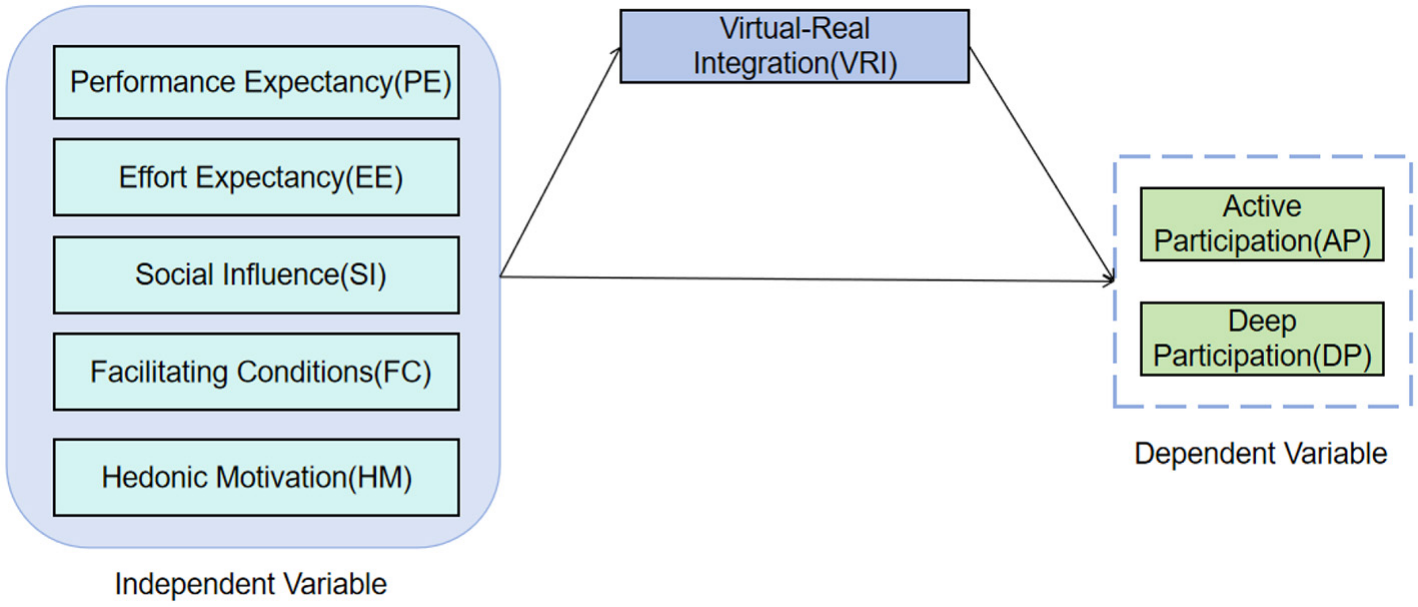
Final research model diagram.

Based on the aforementioned hypotheses, the theoretical model constructed in this study is shown in Figure 1. This model depicts how the five core independent variables—performance expectancy, effort expectancy, social influence, facilitating conditions, and hedonic motivation—influence the two pathways of active sports participation and deep sports participation through the mediating variable virtual-real integration. Simultaneously, the model also incorporates gender, age, and grade as control variables to test the robustness of the relationships between the core variables after excluding the potential interference of these demographic factors. The rationale for selecting these control variables is as follows: existing research indicates that adolescents of different genders and age groups may exhibit differences in technology acceptance and sports participation behavior (Angosto et al., 2023; Zhang et al., 2023; Wang et al., 2022). Furthermore, grade level is often associated with academic pressure and discretionary time, which may also affect the depth and frequency of their sports participation.

## 3 Method

### 3.1 Scale development and validation

In this research model, the independent variable is the acceptance and utilization of digital sports technology, the dependent variable is adolescent sports participation, and the mediating variable is virtual-real integration. Control variables include gender, age, and grade level. All items, excluding control variables, employ a 5-point Likert scale (1 = “Strongly Disagree” to 5 = “Strongly Agree”), with higher total scores indicating greater levels of the corresponding construct. To ensure the scientific rigor and validity of the measurement instruments, all scales underwent rigorous theoretical development and multiple rounds of revision.

#### 3.1.1 Scale development process

This study comprises three core scales, each developed in accordance with a systematic questionnaire development paradigm. The independent variable scale was adapted from the established UTAUT2 scale, undergoing rigorous contextualization. Both dependent and mediating variable scales employed a hybrid development approach combining theoretical construction with empirical validation. Initially, grounded inpersonal initiative framework (Frese et al., 1997), flow theory (Csikszentmihalyi, 1997; Jackson and Marsh, 1996), and technological affordances theory (Gibson, 1979; Du et al., 2019), an initial item pool was formed through literature review. Subsequently, through in-depth field observations and contextualized participant-led interviews, a two-week study involving 13 adolescents was conducted to gather authentic behavioral and psychological perception data, ensuring high ecological validity of the items. This formed the preliminary versions of each scale.

To preliminarily assess the scales' reliability and applicability, a pre-survey was first administered to 59 adolescents. Through reliability analysis and item analysis, unsuitable items were removed. Final results demonstrated excellent overall reliability for the Digital Sports Technology Acceptance and Usage Scale, with a Cronbach's alpha coefficient of 0.978. The virtual-real integration Scale also exhibited outstanding reliability, achieving an alpha coefficient of 0.979, while the Sports Participation Scale recorded an alpha coefficient of 0.938. These pre-test results indicated excellent internal consistency for the initial versions of all scales and sound item design.

Following these positive pre-test findings, the scales entered formal revision and validation. All scales underwent expert content validity review, with minor adjustments made to certain item phrasing. Comprehensive reliability and validity testing was subsequently conducted using a large formal sample. The finalized scales achieved excellent standards in reliability, convergent validity, and discriminant validity, providing a robust measurement foundation for structural equation modeling analysis.

#### 3.1.2 Scale reliability and validity

The Digital Sports Technology Acceptance and Usage Scale comprises five dimensions: performance expectancy, effort expectancy, social influence, facilitating conditions, and hedonistic motivation, totalling 17 items. The overall Cronbach's alpha coefficient in formal testing was 0.893, with dimension-specific alphas ranging from 0.811 to 0.903, demonstrating sound reliability stability. Composite reliability ranged from 0.783 to 0.896, exceeding Bagozzi et al.'s recommended threshold of 0.6 (Bagozzi and Yi, 1988). The extracted mean variance exceeded 0.50, meeting Fornell et al.'s criterion of ≤ 0.5, thereby supporting convergent validity. Distinctiveness validity analysis revealed that the square root of the average variance extracted for each factor exceeded the maximum correlation coefficient between that factor and others. Furthermore, the heteroscedasticity-monotonicity ratio for any pair of variables remained below the 0.85 threshold (Henseler et al., 2015). Confirmatory factor analysis results indicated that the proposed five-factor model demonstrated good fit.

The Sports Participation Scale comprises two dimensions—Active Participation and Deep Participation—with six items in total. In formal testing, the scale yielded an overall Cronbach's alpha coefficient of 0.813, with dimension-specific coefficients of 0.838 and 0.821 respectively. The composite reliability coefficients for the two dimensions were 0.844 and 0.825 respectively, with average variance extracted (AVE) values of 0.644 and 0.612, supporting good convergent validity. Distinctive validity indicators also met standards, with the heterogeneity-monotonicity ratio between dimensions below 0.85. Confirmatory factor analysis confirmed the two-factor model exhibited excellent fit.

The Virtual-Real Integration Scale, as a self-developed scale for the mediating variable, consists of 4 items. During formal testing, the scale exhibited an overall Cronbach's alpha coefficient of 0.817. All item factor loadings exceeded acceptable thresholds, with composite reliability at 0.822 and average variance extracted at 0.548. Confirmatory factor analysis indicated model fit indices met standards, confirming the scale possesses adequate reliability and construct validity for subsequent analysis.

The reliability and validity test results for scales are presented in Tables 1–4.

**Table 1:** Reliability and convergent validity analysis results.

**Scale**	**Factor**	**Item**	**Standardized loading**	**Cronbach's α**	**CR**	**AVE**
Digital sports technology acceptance and usage	Performance Expectancy (PE)	PE1	0.841	0.874	0.882	0.716
		PE2	0.820			
		PE3	0.827			
	Effort Expectancy (EE)	EE1	0.852	0.903	0.896	0.684
		EE2	0.770			
		EE3	0.748			
		EE4	0.879
	Social influence (SI)	SI1	0.838	0.811	0.783	0.547
		SI2	0.844			
		SI3	0.810			
	Facilitating conditions (FC)	FC1	0.875	0.888	0.830	0.550
		FC2	0.863			
		FC3	0.863			
		FC4	0.866
	Hedonic motivation (HM)	HM1	0.833	0.825	0.838	0.633
		HM2	0.741			
		HM3	0.832			
Sports participation	Active participation (AP)	AP1	0.846	0.838	0.844	0.644
		AP2	0.830			
		AP3	0.874			
	Deep participation (DP)	DP1	0.851	0.821	0.825	0.612
		DP2	0.869			
		DP3	0.826			
Virtual-real integration	Virtual-real integration (VRI)	VRI1	0.824	0.817	0.822	0.548
		VRI2	0.876			
		VRI3	0.831			
		VRI4	0.665			

**Table 2:** Discrimination validity tests results.

**Factor**	**PE**	**EE**	**SI**	**FC**	**HM**	**AP**	**DP**	**VRI**
PE	0.839							
EE	0.428	0.841						
FC	0.324	0.360	0.769					
SI	0.312	0.417	0.254	0.816				
HM	0.380	0.401	0.389	0.298	0.782			
AP	0.482	0.474	0.414	0.348	0.444	0.796		
DP	0.341	0.397	0.440	0.321	0.395	0.390	0.778	
VRI	0.441	0.479	0.418	0.508	0.460	0.506	0.468	0.745

**Table 3:** HTMT results.

**Factor**	**PE**	**EE**	**SI**	**FC**	**HM**	**AP**	**DP**	**VRI**
PE	1.000							
EE	0.483	1.000						
FC	0.385	0.421	1.000					
SI	0.354	0.465	0.300	1.000				
HM	0.447	0.464	0.474	0.348	1.000			
AP	0.563	0.545	0.503	0.403	0.534	1.000		
DP	0.402	0.462	0.538	0.376	0.480	0.470	1.000	
VRI	0.527	0.559	0.518	0.599	0.565	0.617	0.576	1.000

**Table 4:** Confirmatory factor analysis (CFA) model fit indices.

**Models**	**χ^2^/*df***	**TLI**	**CFI**	**RMSEA**	**SRMR**
Digital sports technology acceptance and usage	1.325	0.975	0.980	0.040	0.043
Sports participation	1.094	0.997	0.999	0.021	0.027
Virtual-real integration	1.529	0.990	0.997	0.050	0.022

### 3.2 Participants

Data were collected using a combination of online and offline methods. The online survey was distributed via the “Questionnaire Star”' platform, while the offline questionnaires were administered on-site during school physical education classes and youth sports club activities. All items were closed-ended questions, completed independently by the respondents. A total of 430 questionnaires were collected. After excluding 32 invalid questionnaires due to patterned responses or internal inconsistencies, 417 valid questionnaires were retained, yielding a valid response rate of 96.98%. The sample size meets the recommendation for structural equation modeling that the sample size should be at least 10 times the number of items (Wu, 2009), as this study utilized 30 items. Among the valid samples, males accounted for 49.4% (206 participants), and females accounted for 50.5% (211 participants). The age distribution was concentrated in two groups: 12–15 years old (junior high school, 51.6%) and 16–18 years old (high school, 45.4%). The sample covered students from regular classes, sports specialty classes, and extracurricular sports clubs, indicating a diverse and representative sample of the adolescent population that is suitable for subsequent empirical analysis.

### 3.3 Data analysis

This study employs Structural Equation Modeling (SEM) to test the theoretical model. SEM can simultaneously handle multiple dependent variables and permits measurement error in both independent and dependent variables, making it suitable for examining the theoretical model in this research, which encompasses multiple latent variables and their complex causal relationships. Specifically, the Bootstrap mediation effect testing method was employed (Preacher and Hayes, 2008). Through repeated sampling (set at 5,000 iterations), confidence intervals for the mediation effect were generated to examine the mediating role of virtual real integration between digital sports technology acceptance and sports participation. A mediation effect was deemed significant when the 95% confidence interval did not include zero.

The data analysis process comprised two stages: firstly, foundational statistical procedures including descriptive statistics, reliability testing, and correlation analysis were conducted using SPSS software; secondly, confirmatory factor analysis and structural equation modeling were performed using AMOS software to evaluate the measurement model's reliability and validity, alongside the structural model's path coefficients and goodness-of-fit. All analyses employed two-tailed tests with a significance level set at α= 0.05.

## 4 Data analysis and results verification

### 4.1 Correlation analysis

**Figure 2:**
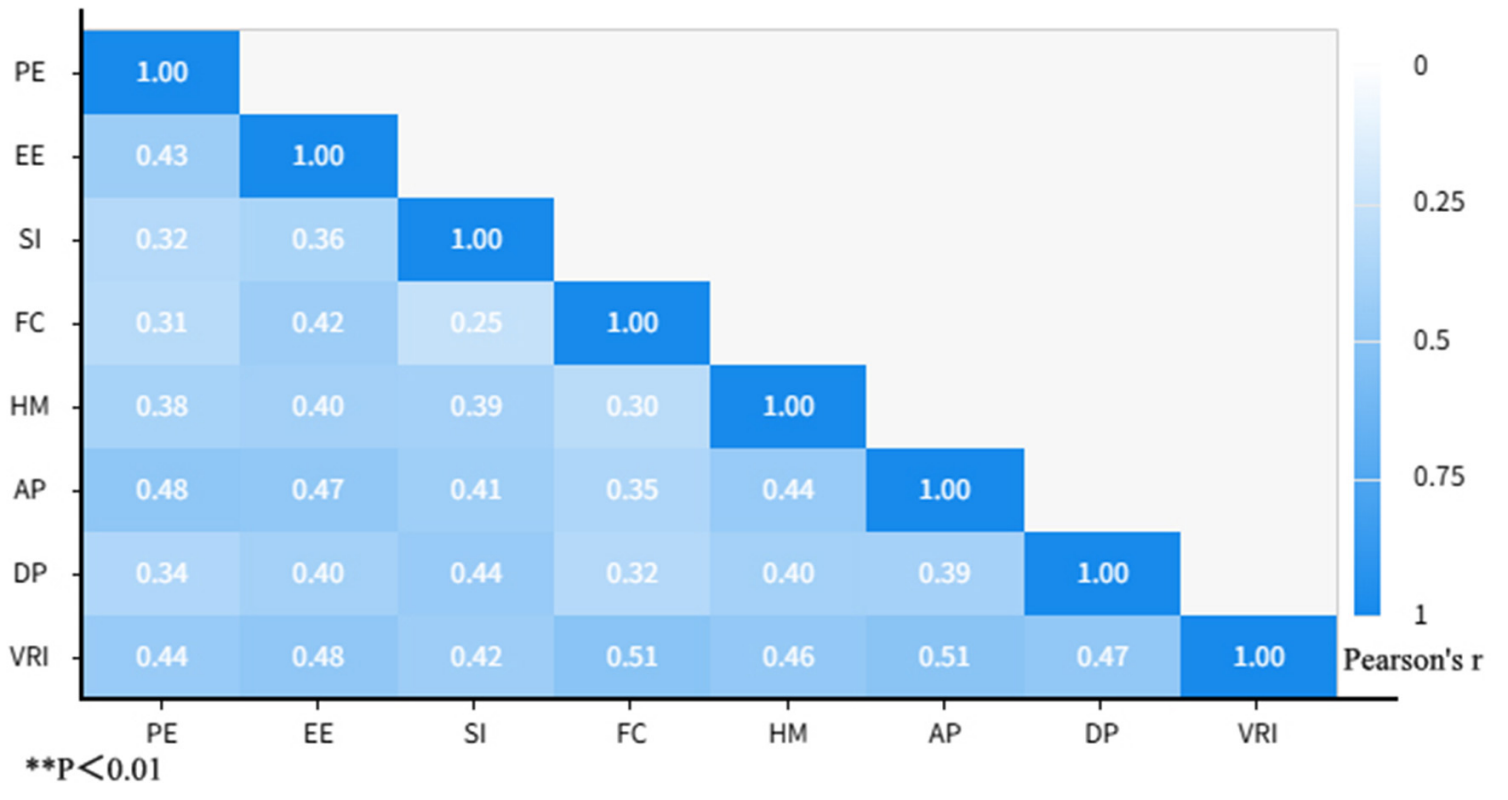
Correlation analysis results.

The results of the correlation analysis (Figure 2) revealed significant positive relationships among all core variables in this study (*p* < 0.01), providing preliminary support for the theoretical hypotheses. Specifically, the five antecedent variables of UTAUT2—performance expectancy, effort expectancy, social influence, facilitating conditions, and hedonic motivation—were significantly and positively correlated with the mediating variable virtual–real integration as well as with the outcome variables active participation and deep participation. Among these, facilitating conditions showed the strongest correlation with virtual–real integration (*r* = 0.508), while performance expectancy exhibited the strongest correlation with active participation (*r* = 0.482). The mediating variable virtual–real integration also demonstrated moderately strong positive correlations with both active participation (*r* = 0.506) and deep participation (*r* = 0.468), preliminarily confirming its bridging role in the model. Furthermore, none of the correlation coefficients between variables exceeded 0.85, indicating that while the constructs are related, they maintain sufficient discriminant validity to meet the requirements for further analysis using structural equation modeling.

### 4.2 Common method bias test

To systematically assess the potential influence of common method bias, this study employed two complementary statistical approaches for testing. First, a more rigorous multilevel confirmatory factor analysis was conducted for nested model comparison. The results are presented in Table 5. The baseline eight-factor model demonstrated the best model fit (χ^2^/df = 1.411, RMSEA = 0.031, CFI = 0.980, TLI = 0.977). As factors were successively constrained to merge in the alternative models, all fit indices showed significant and systematic deterioration. Notably, the model in which all variables were combined into a single factor yielded a very poor fit (χ^2^/df = 9.349, RMSEA = 0.142, CFI = 0.560, TLI = 0.524), indicating that common method variance cannot account for the majority of the covariation among the constructs. Second, the Harman's single-factor test (Podsakoff et al., 2003) was performed for cross-validation. When all measurement items were subjected to exploratory factor analysis, eight factors exhibited eigenvalues greater than 1, and the first factor explained only 35.72% of the total variance, which is below the 40% threshold. Based on the combined results of these statistical tests, it can be concluded that common method bias does not pose a serious threat to the data quality or the core findings of this study.

**Table 5:** Results of multilevel confirmatory factor analysis.

**Model**	**χ^2^**	**df**	**χ^2^/df**	**RMSEA**	**SRMR**	**CFI**	**TLI**
Eight-Factor Model: PE, EE, SI, FC, HM, VRI, AP, DP	417.644	296.000	1.411	0.031	0.0452	0.980	0.977
Seven-factor model: PE, EE, SI, FC, HM, VRI, AP + DP	751.778	303.000	2.481	0.060	0.0568	0.927	0.916
Six-factor model: PE, EE, SI, FC, HM, VRI + AP + DP	967.463	309.000	3.131	0.072	0.0549	0.893	0.878
Five-factor model: PE + EE, SI, FC, HM, VRI + AP + DP	1, 535.067	314.000	4.889	0.070	0.0807	0.802	0.778
Four-factor model: PE + EE, SI + FC, HM, VRI + AP + DP	2, 003.705	318.000	6.301	0.113	0.1150	0.726	0.698
Three-factor model: PE + EE, SI + FC + HM, VRI + AP + DP	2, 446.920	321.000	7.623	0.126	0.121	0.654	0.622
Two-factor model: PE + EE + SI + FC + HM, VRI + AP + DP	2, 879.924	323.000	8.916	0.138	0.102	0.584	0.548
One-factor model: PE + EE + SI + FC + HM, VRI + AP + DP	3,028.976	324.000	9.349	0.142	0.103	0.560	0.524

*N* = 417; “+” indicates factor merging. PE, performance expectancy; EE, effort expectancy; SI, social influence; FC, facilitating conditions; HM, hedonic motivation; VRI, virtual–real integration; AP, active participation; DP, deep participation.

### 4.3 Gender-based measurement invariance test

This study focuses on the complete influence mechanism of technology acceptance- virtual-real integration-sports participation. Testing the invariance of the integrated model allows for more direct verification of whether the measurement structure of the entire theoretical framework remains stable across different gender groups. Therefore, prior to conducting structural equation modeling, this research performed cross-gender measurement invariance tests on the model.

The results (Table 6) indicate that all levels of invariance (configurational, weak, strong, and strict) in the integrated model meet psychometric standards (Δ CFI ≤ 0.001, Δ RMSEA ≤ 0.002). This confirms that measurement parameters exhibit no significant differences between gender groups. This indicates that the core variables and their measurement relationships in this study are consistent across male and female samples.

**Table 6:** Coefficient table for measurement invariance tests.

**Model**	**χ^2^**	***df***	**χ^2^/*df***	**CFI**	**RMSEA**	**ΔCFI**	**ΔRMSEA**
1	746.002^***^	592	1.260	0.975	0.025		
2	759.855^***^	611	1.244	0.976	0.024	0.001	–0.001
3	786.213^***^	638	1.232	0.976	0.024	0.000	0.000
4	845.457^***^	701	1.206	0.977	0.022	0.001	–0.002

***p < 0.001.

### 4.4 Model fit

To examine the fit between the theoretical model and the empirical data, this study utilized AMOS software to conduct a comprehensive evaluation of the overall model fit using multiple indices: absolute, incremental, and parsimonious fit.

The analysis results showed that for the absolute fit indices, the χ^2^/df value was 1.380 (meeting the criterion of < 3); the RMSEA was 0.030 (better than the critical value of 0.08). The incremental fit indices were excellent, with TLI = 0.973 and CFI = 0.977 (both exceeding 0.9). The parsimonious fit indices, PNFI = 0.789 and PCFI = 0.836, were both significantly higher than the benchmark of 0.5. All fit indices reached or surpassed the recommended thresholds, indicating an ideal match between the theoretical model and the observed data. The overall structure of the model is reasonable and suitable for subsequent path analysis and hypothesis testing. The results are shown in Table 7.

**Table 7:** Fit indices for the structural equation mode.

**Fit index**	**Recommended value**	**Obtained value**
χ^2^/df	< 3.0	1.380
RMSEA	< 0.08	0.030
TLI	>0.9	0.973
CFI	>0.9	0.977
PNFI	>0.5	0.789
PCFI	>0.5	0.836

### 4.5 Hypothesis testing results

Based on the path analysis results obtained through the AMOS structural equation modeling, this study examined the proposed theoretical hypotheses. First, the test results concerning the control variables revealed that only age exerted a statistically significant positive effect on the mediating variable virtual-real integration(β= 0.089, *p* < 0.05). In contrast, gender and grade showed no significant impact on virtual-real integration, nor did any of the control variables significantly affect the dependent variables active participation and deep participation (*p*> 0.05). These findings indicate that the potential effects of demographic variables have been incorporated and statistically isolated in the model, with no significant interference detected in the relationships among the core constructs. Consequently, the subsequent analytical conclusions maintain a high degree of purity and reliability.

**Figure 3:**
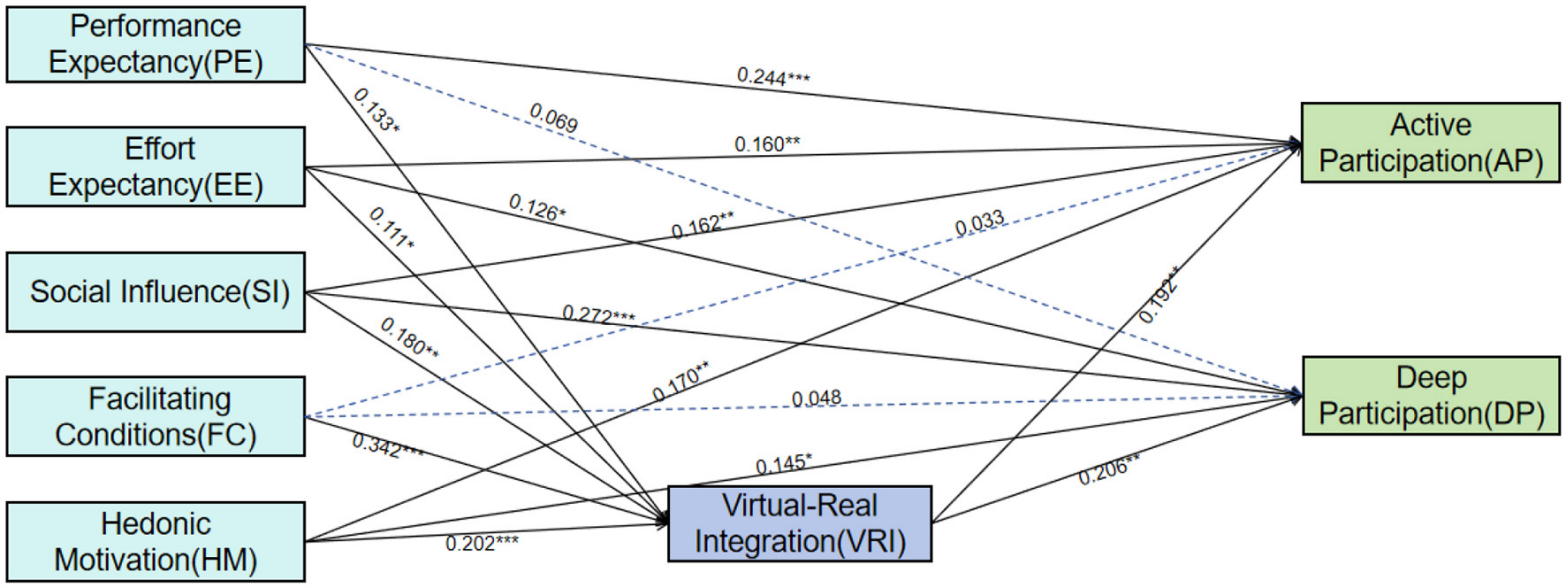
Continuous use intention model based on UTAUT2. ^***^p < 0.001, ^**^p < 0.01, ^*^p < 0.05. Solid lines denote supported hypotheses; dashed lines denote unsupported hypotheses.

Regarding the core hypotheses, the results showed that the direct path from performance expectancy to deep sports participation was not significant (β= 0.069, *p*> 0.05), thus, hypothesis H1b was not supported. Additionally, the direct effects of facilitating conditions on both active and deep sports participation were not statistically significant (β= 0.033, *p*>0.05; β= 0.048, *p*>0.05). Therefore, hypotheses H4a and H4b were also not supported. (The standardized model is shown in Figure 3, and the model path results are presented in Table 8).

**Table 8:** Model path testing results.

**Dependent Variable**	**Independent Variable**	**Hypothesis**	**Unstandardized Path Coefficient**	**Standardized Path Coefficient**	**S.E**.	**C.R**.	***P***	***R* ^2^**	**Result**
AP	PE	H1a	0.156	0.244	0.036	4.363	^***^	0.515	Accepted
	EE	H2a	0.129	0.160	0.046	2.798	^**^		Accepted
	SI	H3a	0.144	0.162	0.053	2.723	^**^		Accepted
	FC	H4a	0.030	0.033	0.052	0.578	0.563		Unaccepted
	HM	H5a	0.152	0.170	0.056	2.729	^**^		Accepted
	VRI	H6a	0.157	0.192	0.057	2.744	^**^		Accepted
DP	PE	H1b	0.045	0.069	0.038	1.173	0.241	0.431	Unaccepted
	EE	H2b	0.103	0.126	0.050	2.054	^*^		Accepted
	SI	H3b	0.249	0.272	0.060	4.171	^***^		Accepted
	FC	H4b	0.045	0.048	0.057	0.781	0.435		Unaccepted
	HM	H5b	0.133	0.145	0.061	2.184	^*^		Accepted
	VRI	H6b	0.172	0.206	0.063	2.738	^**^		Accepted
VRI	PE	H1c	0.104	0.133	0.042	2.476	^*^	0.507	Accepted
	EE	H2c	0.109	0.111	0.055	1.971	^*^		Accepted
	SI	H3c	0.196	0.180	0.063	3.129	^**^		Accepted
	FC	H4c	0.381	0.342	0.061	6.259	^***^		Accepted
	HM	H5c	0.221	0.202	0.066	3.376	^***^		Accepted

^***^p < 0.001, ^**^p < 0.01, ^*^p < 0.05.

All other hypotheses were supported. Specifically, the factors exerting a significant positive influence on active sports participation, ranked by effect size, were: performance expectancy (β= 0.244), hedonic motivation (β= 0.170), effort expectancy (β= 0.160), social influence (β= 0.162), and the degree of virtual-real integration (β= 0.192). The factors significantly influencing deep sports participation were: social influence (β= 0.272), the degree of virtual-real integration (β= 0.206), effort expectancy (β= 0.126), and hedonic motivation (β= 0.145). Performance expectancy, effort expectancy, social influence, facilitating conditions, and hedonic motivation all demonstrated significant positive effects on the degree of virtual-real integration, with the effect of facilitating conditions being the most prominent (β= 0.342). The model exhibited strong explanatory power for the three core endogenous variables. The squared multiple correlations (R^2^) for virtual-real integration, active participation, and deep participation were 0.507, 0.515, and 0.431, respectively. This indicates that the antecedent variables (performance expectancy, effort expectancy, social influence, facilitating conditions, and hedonic motivation) collectively accounted for 50.7% of the variance in VRI, and that VRI, together with the other antecedent variables, explained 51.5% and 43.1% of the variance in AP and DP, respectively.

### 4.6 Mediation effect test results

Based on the Bootstrap mediation effect test results (Hopwood, 2007) (Table 9), an analysis of the mediating role of the virtual-real integration in the adolescent sports participation model yielded the following conclusions: the mediation effect analysis indicates that the virtual-real integration played a significant mediating role in the relationships between performance expectancy, effort expectancy, social influence, facilitating conditions, hedonic motivation, and the two types of sports participation behaviors. The specific results are as follows:

**Table 9:** Mediation effect test results.

**Hypotheses**	**Path**	**Effect**	**β**	**S.E**.	***P***	**95% BootLLCI**	**95% BootULCI**
H7a	PE → VRI → AP	Indirect	0.026	0.015	0.009	0.005	0.067
		Direct	0.244	0.054	0.000	0.138	0.193
		Total	0.269	0.054	0.000	0.163	0.374
H8a	EE → VRI → AP	Indirect	0.021	0.014	0.031	0.002	0.059
		Direct	0.160	0.057	0.006	0.043	0.249
		Total	0.182	0.057	0.003	0.065	0.287
H9a	SI → VRI → AP	Indirect	0.035	0.018	0.003	0.009	0.082
		Direct	0.162	0.063	0.010	0.036	0.415
		Total	0.196	0.061	0.002	0.078	0.314
H10a	FC → VRI → AP	Indirect	0.066	0.030	0.004	0.019	0.137
		Direct	0.033	0.056	0.526	–0.079	0.176
		Total	0.099	0.050	0.053	–0.001	0.197
H11a	HM → VRI → AP	Indirect	0.039	0.020	0.003	0.010	0.091
		Direct	0.170	0.059	0.004	0.051	0.279
		Total	0.209	0.056	0.000	0.097	0.317
H7b	PE → VRI → DP	Indirect	0.027	0.016	0.010	0.005	0.070
		Direct	0.069	0.064	0.280	–0.059	0.350
		Total	0.096	0.063	0.134	–0.032	0.216
H8b	EE → VRI → DP	Indirect	0.023	0.015	0.034	0.002	0.063
		Direct	0.126	0.064	0.052	–0.003	0.269
		Total	0.149	0.064	0.024	0.020	0.270
H9b	SI → VRI → DP	Indirect	0.037	0.018	0.005	0.010	0.083
		Direct	0.272	0.073	0.001	0.128	0.286
		Total	0.309	0.070	0.000	0.170	0.445
H10b	FC → VRI → DP	Indirect	0.071	0.029	0.006	0.020	0.133
		Direct	0.048	0.067	0.495	–0.085	0.140
		Total	0.119	0.063	0.075	–0.012	0.234
H11b	HM → VRI → DP	Indirect	0.042	0.020	0.004	0.012	0.094
		Direct	0.145	0.069	0.034	0.013	0.285
		Total	0.187	0.068	0.005	0.053	0.318

In the active participation in sports pathway, all five antecedent variables produced significant indirect effects (*p* < 0.05; 95% CIs included zero) through the degree of virtual-real integration. Furthermore, with the exception of facilitating conditions, the direct effects of the other variables on active participation were also significant. The direct effect of facilitating conditions was not significant (β = 0.033, *p* = 0.526), indicating that its influence is entirely mediated by the degree of virtual-real integration. That is, facilitating conditions promote active participation only by enhancing virtual-real integration, with no direct effect present.

In the path predicting deep participation, the direct effects of both performance expectancy (β = 0.069, *p* = 0.280) and facilitating conditions (β = 0.048, *p* = 0.495) were not statistically significant. The direct effect of effort expectancy approached significance (β = 0.126, *p* = 0.052), suggesting a potential direct promoting effect, although the statistical evidence for this path is weaker than that for other significant paths. Effort expectancy, social influence, and hedonic motivation each exhibited either significant or marginally significant direct effects, while also demonstrating significant indirect effects mediated by virtual-real integration.

**Figure 4:**
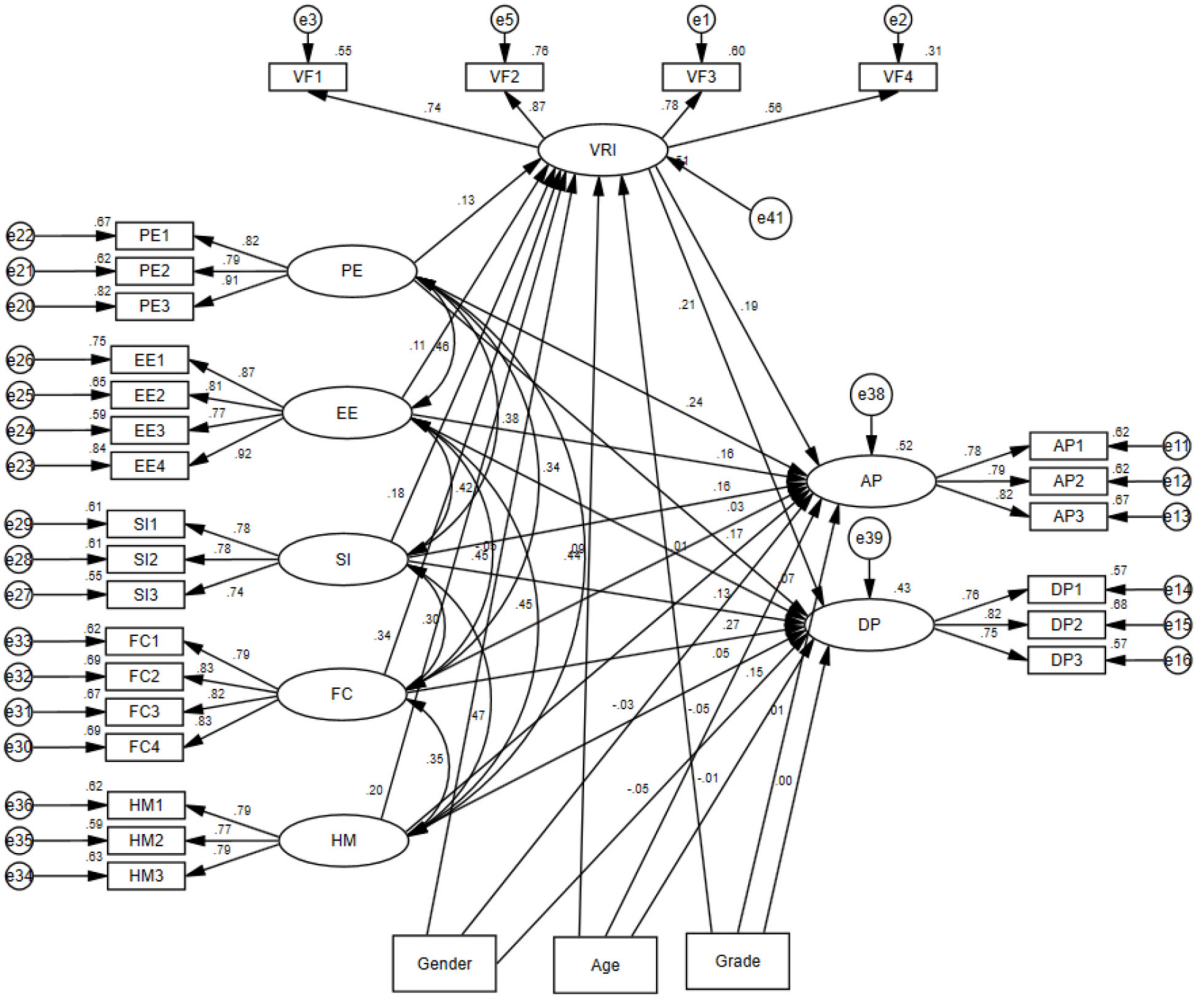
Assuming standardized output results of the model.

Regarding the total effects, the Bootstrap results indicated that the total effects of performance expectancy and facilitating conditions on deep sports participation were not statistically significant (*p* = 0.134 and *p* = 0.075; 95% CIs included zero). In contrast, the total effects of all other antecedent variables on both active and deep participation were significant (*p* < 0.05), with the exception of the total effect of facilitating conditions on active participation, which was marginally significant (*p* = 0.053). Overall, these results indicate that the integrated model possesses good predictive power for explaining adolescents' digital sports participation behaviors. The final path coefficients of the model are shown in Figure 4.

## 5 Research findings and discussion

### 5.1 Pathways and differentiated effects of core influencing factors

Based on the Unified Theory of Acceptance and Use of Technology (UTAUT2), this study constructed a theoretical model to analyze adolescents' digital sports participation behavior. Based on structural equation modeling of questionnaire data from 417 adolescents, this study systematically investigated the influence of five core variables (performance expectancy, effort expectancy, social influence, facilitating conditions, and hedonic motivation) on adolescents' active and deep sports engagement, as mediated by the key construct of virtual-real integration. Findings indicate that the core constructs of the UTAUT2 model possess general explanatory power in adolescent digital sports participation contexts, yet their influence pathways and effectiveness exhibit significant differentiation.

#### 5.1.1 The mediated role of performance expectancy

Performance expectancy exerted a significant direct positive effect on active participation in exercise (β = 0.244, *p* < 0.001), but its direct path to deep engagement in exercise failed to reach statistical significance (β = 0.069, *p* = 0.241). However, performance expectations exerted a significant positive effect on virtual-real integration (β = 0.133, *p* < 0.05) and produced a significant indirect effect on deep engagement in sports through this mediating variable. This indicates that adolescents' expectations regarding digital sports technology enhancing athletic performance primarily drive their exploratory and planned active participation behaviors. For deep engagement behaviors requiring higher emotional and cognitive investment, the influence of performance expectations is fully realized through promoting the integration of online and offline sports activities. In other words, only when adolescents perceive the technology as useful and are consequently more willing to combine technological data with offline training can their sports experience ultimately be deepened.

#### 5.1.2 The foundational driving role of effort expectancy

Effort expectancy exerts a significant direct positive influence on active participation (β = 0.160, *p* < 0.01), deep engagement (β = 0.126, *p* < 0.05), and virtual-real integration (β = 0.111, *p* < 0.05), with partial mediating effects observed. This indicates that technological ease of use and masterability serve as foundational prerequisites for stimulating adolescents' willingness to participate. Low technical barriers not only directly encourage adolescents to engage in sports more frequently and with greater immersion but also reduce behavioral resistance to integrating digital tools with physical exercise.

#### 5.1.3 The strong driving effect of social influence

Social influence stands as one of the variables with the most prominent effect size in the model, exhibiting a particularly significant direct impact on deep sports participation (β = 0.272, *p* < 0.001). Simultaneously, it significantly influenced both active participation (β = 0.162, *p* < 0.01) and virtual-real integration (β = 0.180, *p* < 0.01). This finding powerfully demonstrates that in adolescents' sports participation decisions, the attitudes and behavioral modeling of significant others—such as peers, parents, and teachers—constitute a potent social force. This aligns with the central role of subjective norms in (Ajzen 1991) Theory of Planned Behavior, where social norms exert a significant influence on behavioral intent that transcends partial intrinsic motivation. A positive community environment not only directly drives adolescents toward deeper engagement in sports but also motivates them to more actively integrate digital technology into their daily physical activities.

#### 5.1.4 The fully mediated role of facilitative conditions

A key finding is that the direct effects of facilitation conditions on active participation (β = 0.033, *p* = 0.563) and deep participation (β = 0.048, *p* = 0.435) were not significant. However, its impact on virtual-real integration was exceptionally strong (β = 0.342, *p* < 0.001), exhibiting complete mediation effects in both pathways. This implies that external resources-such as hardware equipment, technical compatibility, and operational support-do not directly enhance adolescents' motivation to exercise. Instead, their value lies in empowering individuals and lowering barriers to integration behavior by making the blending of virtual and real worlds feasible and seamless, thereby indirectly promoting participation. This finding provides strong empirical support for prioritizing improvements to digital sports infrastructure and support environments in practice.

#### 5.1.5 The intrinsic core value of hedonic motivation

Hedonic motivation plays a significant driving role in adolescents' participation in digital sports. Research findings indicate that hedonic motivation not only exerts a significant direct positive influence on active participation (β = 0.170, *p* < 0.01) but also exerts a significant direct influence on deep engagement (β = 0.145, *p* < 0.05). This finding supports the widely held perspective in information systems research that hedonic emotions can directly trigger users' willingness to use (Zhang et al., 2023). Furthermore, hedonic motivation exerts significant indirect effects on both active participation and deep engagement by enhancing the virtual-real integration level. This indicates that the enjoyment provided by digital technologies serves not only as a direct attraction but also as a crucial “catalyst”—motivating adolescents to actively integrate virtual experiences with offline sports activities in pursuit of and to sustain the pleasure and satisfaction derived from online interactions (Vorderer et al., 2004). It is precisely this pleasure-driven, virtual-physical hybrid behavior pattern that ultimately transforms participation from a transient, random trial into a long-term, sustained, and deeply committed state (Tang et al., 2020). Thus, the pleasure motive serves as the core intrinsic driver propelling adolescents from willingness to try to deep immersion.

### 5.2 The key mediating role of virtual-real integration

The core contribution of this study's theoretical model lies in establishing and validating the central mediating mechanism of virtual-real integration between technology acceptance and sports participation behavior. Mediating effect tests indicate that, while facilitating conditions exert their influence entirely through this variable, the impact of performance expectations and effort expectations on deep engagement is also fully mediated by it. Hedonic motivation and community influence exert their effects partially through this variable.

This finding reveals the intrinsic logic of adolescent digital sports participation: technological factors (performance expectancy, effort expectancy and facilitating conditions) and psychosocial factors (social influence, hedonic motivation) first jointly influence the breadth and depth of adolescents' connections between virtual spaces and real-world sports. This behavioral pattern of virtual-real integration then becomes the direct driving force stimulating their active participation and deep engagement. The degree of virtual-real integration thus forms a crucial bridge connecting technological acceptance willingness with actual sports behavior, explaining the internal process by which willingness translates into action.

### 5.3 Theoretical contributions and practical implications

In terms of theoretical contributions, this study offers three key insights: First, it successfully applies the UTAUT2 model to the emerging field of youth digital sports, validating the framework's applicability and explanatory power in this context. Second, it proposes and validates the key mediating construct of virtual-real integration, offering a mechanistic explanation for how technology acceptance translates into deep sports engagement. This addresses the limitations of classical technology acceptance models in explaining the intention-to-behave conversion mechanism. Third, it reveals significant differentiation in the influence pathways of different antecedent variables on sports participation behavior, providing a more refined theoretical perspective for future research.

In terms of practical implications, this study offers specific recommendations for different stakeholders: For technology developers, focus should be placed on designing features that naturally bridge online feedback with offline exercise, such as automatically generating training plans based on exercise data or setting up virtual achievements that unlock physical rewards. For schools and physical education professionals, leveraging community influence is crucial. This can be achieved by organizing blended online-offline sports challenges and establishing exercise data-sharing communities to foster positive social comparison and mutual motivation among adolescents. For policymakers, promoting youth sports participation requires more than providing hardware. Greater emphasis should be placed on training and support to help adolescents effectively integrate digital tools with physical activities, rather than merely offering access opportunities.

### 5.4 Research limitations and future directions

This study has the following limitations: First, the cross-sectional design limits the strength of causal inference. Future research could employ longitudinal tracking or experimental designs to further validate causal relationships between variables. Second, the sample originates from a specific region, and the generalizability of conclusions requires testing across different cultural contexts. Additionally, the measurement of virtual-real integration remains open to refinement. Future work could employ multi-method validation by integrating objective behavioral data (e.g., app usage logs, motion sensor data). Finally, this study simplified the UTAUT2 model for contextualization. Future research involving broader populations or mature application scenarios may consider reintroducing variables such as price value and habit to test the model's completeness and boundary conditions.

## Data Availability

The raw data supporting the conclusions of this article will be made available by the authors, without undue reservation.
